# Healthcare-seeking behavior, treatment delays and its determinants among pulmonary tuberculosis patients in rural Nigeria: a cross-sectional study

**DOI:** 10.1186/1472-6963-13-25

**Published:** 2013-01-17

**Authors:** Kingsley N Ukwaja, Isaac Alobu, Chibueze O Nweke, Ephraim C Onyenwe

**Affiliations:** 1 Department of Internal Medicine, Federal Teaching Hospital, Abakaliki, Ebonyi State, Nigeria; 2 National Tuberculosis and Leprosy Control Programme, Ministry of Health, Abakaliki, Ebonyi State, Nigeria; 3 Department of Family Medicine, Federal Teaching Hospital, Abakaliki, Ebonyi State, Nigeria

**Keywords:** Tuberculosis, Health-seeking delays, Private sector, Public sector, Rural, Low-resource setting

## Abstract

**Background:**

Nigeria ranks fourth among 22 high tuberculosis (TB) burden countries. Although it reached 99% DOTS coverage in 2008, current case detection rate is 40%. Little is known about delays before the start of TB therapy and health-seeking behaviour of TB patients in rural resource-limited settings. We aimed to: 1) assess healthcare-seeking behaviour and delay in treatment of pulmonary TB patients, 2) identify the determinants of the delay in treatment of pulmonary TB.

**Methods:**

We conducted a cross-sectional study of adult new pulmonary TB patients notified to the National Tuberculosis Control Programme (NTP) by three rural (two mission/one public) hospitals. Data on health-seeking and delays were collected using a standardised questionnaire. We defined patient delay as the interval (weeks) between the onset of cough and the first visit to any health provider, and health system delay as the time interval (weeks) between patient's first attendance to any health provider, and the onset of treatment. Total delay is the sum of both delays. Multiple linear regression models using nine exposure variables were built to identify determinants of delays.

**Results:**

Of 450 patients (median age 30 years) enrolled, most were males (55%), subsistent farmers (49%), rural residents (78%); and 39% had no formal education. About 84% of patients reported first consulting a non-NTP provider. For such patients, the first facilities visited after onset of symptoms were drug shops (79%), traditional healers (10%), and private hospitals (10%). The median total delay was 11 (IQR 9–16) weeks, patient delay 8 (IQR 8–12) and health system (HS) delay 3 (IQR 1–4) weeks. Factors associated with increased patient delay were older age (P <0.001) longer walking distance to a public facility (<0.001), and urban residence (P <0.001). Male gender (P = 0.001) and an initial visit to a non-NTP provider (P = 0.025) were independent determinants of prolonged HS delay. Those associated with longer total delay were older age (P <0.001), male gender (P = 0.045), and urban residence (P<0.001).

**Conclusion:**

Overall, TB treatment delays were high; and needs to be reduced in Nigeria. This may be achieved through improved access to care, further education of patients, engagement of informal care providers, and strengthening of existing public-private partnerships in TB control.

## Background

Early diagnosis and prompt treatment remains essential components for effective tuberculosis (TB) control. Delay in TB diagnosis may increase infectivity, worsen the disease state, and enhance the risk of death [1,2]. Since the late 1990s, the coverage of Directly Observed Treatment Short Course (DOTS) has increased in many developing countries, but this was not coupled with a parallel rise in the case detection rate [3]. Besides coverage, several other factors influence timely access of TB patients to appropriate health services. Longer patient delays were found among patients with HIV-infection, sputum smear-negative, and those living in rural/remote settings [4]. A systematic review in Sub-Saharan Africa showed that travel time and consultation with a traditional healer was associated with patient delay [5]. As these factors can vary in different populations and health systems, it is important to evaluate factors affecting patient/health system delay in specific settings.

In the Nigerian health system, services are provided at three levels namely: primary, secondary and tertiary. The local government areas (LGAs) provides the primary level of care; state governments the secondary level of care and provision of technical guidance to the LGAs, and the federal government is responsible for the tertiary level of care as well as policy formulation and technical guidance to the states [6]. The private sector and non-governmental organizations, also provide considerable services at all the levels of health care; accounting for about 50% of health care delivery in the country [6]. From the onset of the National TB Control Programme (NTP), private (not-for-profit) mission health facilities and public (tertiary, secondary and some primary) health institutions have been involved in the TB control programme of the country [6]. Recently, few for-profit private health facilities are been incorporated in to the programme through the public-private mix model [7]. Within the public sector, TB consultations, diagnostic, drugs and hospitalization services are provided free of charge. At the private facilities, TB diagnostic and treatment services are provided free-of-charge. However, all patients irrespective of their health problem visiting the facility pay administrative fees. Following diagnosis, TB patients admitted at the private hospitals are required to pay additional fees for accommodation and feeding.

Currently, Nigeria ranks fourth among 22 countries with a ‘high burden’ of TB and 25% of TB patients are HIV co-infected [8]. Although it reached 99% DOTS coverage in 2008 [6], its current case detection rate is 40% and treatment success rate was 83% in 2009 [8]. The TB epidemic in Nigeria is concentrated in densely populated rural areas [9]. The challenge posed by the low case detection rate can be addressed by studying patient and health system delays in TB care in this population. Two previous studies on delays in TB treatment in Nigeria evaluated patients in urban settings [10,11]. Also, in the last five years, efforts have been made by the NTP to educate the general public about TB [12,13] and to engage the private sector [7]. With these initiatives still ongoing, we aim to describe the healthcare seeking behaviour of adults prior to initiation of anti-tuberculosis treatment at formal public and private NTP facilities in rural Nigeria. We sought to assess health-seeking behaviour and delay in treatment of pulmonary TB patients, and to identify the determinants of the treatment delays.

## Methods

### Study design

A cross-sectional survey of new pulmonary tuberculosis patients starting treatment who were ≥15 years old at three study facilities was conducted from January to September 2011.

### Study setting

The study was conducted in Ebonyi State, in the southeast of Nigeria. It has a population of over 2.5 million people, and 80% of them live in rural areas [14]. Currently, the state has one of the highest unemployment rates in Nigeria (23%) and 74% of its population live below poverty [15]. Furthermore, compared to national figures ($1200), the per capita gross domestic product of Ebonyi state was $198 in 2008/09 [16]. The state has a high notification rate of new TB cases, with 77/100 000 in 2009 [17]. It is divided into three zones having 13 LGAs with 130 healthcare facilities currently providing TB services. TB case finding in the state, as in other places in Nigeria uses the passive case finding strategy [6]. Because of several resource challenges, there is limited availability of public facilities in rural settings in Ebonyi State. Thus, several mission (private) hospitals give primary and secondary care services in those settings.

Three rural hospitals (two private and one public) were chosen as the study sites based on logistics, high TB notification rates and geographical spread [17]. The private (mission) hospitals are Presbyterian Joint Hospital, Uburu and Mile Four Hospital Abakaliki; while the public hospital (secondary health facility) was General Hospital Onueke. The three study facilities accounts for 70% of total TB notification in the state [17], and they each represent the largest hospital for TB services in each of the three zones of the state. TB/HIV collaborative activities exist in all the study facilities; therefore, all TB patients are counselled and tested for HIV, and vice versa.

### Participants’ selection

Participants were newly diagnosed pulmonary TB patients aged ≥15 years, who had commenced treatment for less than two months (i.e. intensive phase of treatment) and are living in Ebonyi state. Eligible patients were invited to participate in the study. Following a written informed consent, consecutive consenting patients were interviewed until the required sample was reached.

### Variables

A pulmonary TB case was defined as an individual newly diagnosed of pulmonary TB, initiated on anti-tuberculosis treatment for less than two months and recorded in the routine clinic TB register. Routine data on sputum smear microscopy, date of treatment initiation, HIV status, smear and type of TB were verified from the TB treatment register and individual patient treatment cards.

The main exposure variable was the type of initial health care provider (HCP) visited after the onset of TB-related symptoms. This was classified as ‘NTP provider’ or ‘non-NTP provider’. A non-NTP provider was defined as any HCP who was not part of the NTP. These included private general practitioners, some primary health centres, traditional healers, and drug shops/pharmacy. A NTP provider was defined as any HCP (public or private) involved in providing TB care and therefore, formally part of the NTP. Other exposure variables include: age, gender, residence, HIV status, smear status, walking distance to the nearest public facility and education.

The main outcome variables were median patient, health system and total delays (in weeks) Patient delay was defined as the interval (number of weeks) between the onset of cough and the first visit to any HCP, while health system delay is the time interval (weeks) between patient's first attendance to any HCP, and the onset of treatment. Total delay is the sum of patient delay and health system delay.

### Data sources / Measurement

A standardised questionnaire adapted from a previous validated WHO questionnaire was used for the survey [3]. The questionnaire included detailed questions about patient's socio-demographic characteristics, the onset of symptoms, health seeking behaviour (including place/category of initial healthcare provider consulted, traditional healers and self-treatment), timing of healthcare seeking steps, and when anti-TB treatment was commenced.

Interviewers were trained for two weeks before the study using practical exercises and were supervised twice weekly by the researchers (ECO, CON) and the district TB control supervisors. The questionnaire was administered in English and Igbo language depending on the patient’s preference. The interviewers translated the questions during the interview using local terminologies agreed upon during the training session. Particular emphasis was given to support the patients in their recall efforts, including the use of a calendar of locally important events and using other probing techniques.

### Sample size

The sample size was calculated using Win Episcope 2.0. With a sample size of 220 patients, we were able to detect an 83% prevalence of patient delay [10], at 95% confidence level and an absolute sampling error of 0.05. However, as it was planned to perform multiple regression analysis, the rule of thumb was used, hence the sample size was doubled to 440 patients. With this sample, our regression models would be stable to assess up to 22 co-variates.

### Data management and analysis

Data were double-entered, cleaned, and analysed using Epi Info (CDC Atlanta, version 3.4.1). Frequency distributions with 95% confidence intervals, and descriptive statistics - including mean/median and interquartile range (IQR), were calculated. Group comparisons were made using the *t*-test for means, χ^2^ test for proportions, Mann–Whitney test for medians in two groups, and the Kruskal-Wallis test for medians in more than two groups. The normality of delay data distribution was assessed using visual inspection of graphs created using the data. Delays were normally distributed. Multiple linear regression models were used to evaluate independent determinants of the outcome variables (patient, health system and total delays). The possible determinants (age, gender, residence, HIV status, smear status, education, walking distance, and type of initial HCP visited after the onset of TB) were assessed for their individual bivariate associations with each type of delay. Known risk factors of delay, and variables associated with delays in the bivariate analysis (*P* ≤0.2) were included in the model. The validity of the models was checked by visually examining the residual errors. Significance tests were two-sided with *P*-values of <0.05 considered statistically significant.

### Ethical approval

Approval for the study was obtained from the Ebonyi State University Teaching Hospital health research and ethics committee.

## Results

### Socio-demographic and clinical characteristics of the respondents

Of 450 patients interviewed with complete records, 354 (79%) were below 40 years old and 246 (55%) were male (Table 1). Most participants live in rural areas 354 (79%), and 276 (61%) had at least six years of formal education. Also, 420 (93%) of them live more than sixty minutes walking distance to the nearest public facility; 348 (77%) patients had acid-fast bacilli in their sputum, and 132 (29%) were HIV seropositive (Table 1). The occupation of majority of the patients were: subsistence farmers 222 (49%), petty traders 114 (25%), casual labourer 54 (12%), housewife 24 (5%), students 24 (5%), and civil servants 12 (2.7%).

**Table 1:** Socio-demographic and clinical characteristics of the study patients (N = 450)

**Variable**	**Total n (%)**
Age (years)	
≤40	354 (79)
41-65	90 (20)
>65	6 (1)
Gender	
Female	204 (45)
Male	246 (55)
Residence	
Rural	354 (79)
Urban	96 (21)
Education	
No formal education	174 (39)
At least 6 years of formal education	276 (61)
Walking distance to the nearest public facility (minutes)	
≤60	30 (7)
61 – 120	138 (31)
>120	282 (62)
Occupation	
Farmers	222 (49)
Trading	114 (25)
Labourer	54 (12)
Housewife	24 (5)
Civil servant	12 (3)
Students	24 (5)
First visited NTP provider	
Yes	72 (16)
No	378 (84)
HIV Status	
Positive	132 (29)
Negative	318 (71)
Smear status	
Smear positive	348 (77)
Smear negative	102 (33)
Had a chest X-ray	
Yes	300 (67)
No	150 (33)

NTP = National Tuberculosis Control Programme; HIV = human immunodeficiency virus.

### Clinical presentation

Most of the patients had cough, night sweats, weight loss and haemoptysis (Table 2). The median duration of cough was 8 weeks, whereas for weight loss, night sweats and haemoptysis it was 4 weeks (Table 2).

**Table 2:** Symptoms* and its duration reported by patients with pulmonary tuberculosis (n = 450)

**Symptoms**	**Symptom**	**Duration (weeks)**
	**N (%)**	**Median (IQR)**
Cough	450 (100)	8 (8, 12)
Fever	300 (66.7)	2 (2, 3)
Weight loss	426 (94.7)	4 (4, 8)
Night sweats	402 (89.3)	4 (4, 8)
Hemoptysis	156 (34.7)	4 (1, 4)
Chest pain	60 (13.3)	2 (2, 4)
Body weakness	138 (30.7)	3 (1, 4)
Others	10 (2.2)	1 (0, 2)

* For most patients, two or more symptoms were reported.

### Healthcare-seeking behaviour

Of the 450 participants, 84% (95%CI 80, 87) reported first going to a non-NTP provider after the onset of TB-related symptoms. Patients who initially went to a non-NTP provider were similar to those who initially went to an NTP provider regarding age (P= 0.4), gender (P =0.5), residence (P = 0.3), educational status (P =0.2), HIV serostatus (P =0.5), and smear status (P = 0.2). Non-NTP providers initially consulted were mainly pharmacy/drug shops (79%), traditional healers (10%) and private clinics (10%); (Table 3). For patients who first visited a non-NTP provider, their main reported reasons for not first visiting a public facility were; because it was too expensive (22%), it takes time (26%), long distance to the facility (23%), belief that they would get better services elsewhere (18%), and mistrust of the public facility (10%); (Table 3).

**Table 3:** Type of non-NTP providers initially visited and reasons for not consulting a public facility first after the onset of TB (n = 378)

**Variable**	**N (%)**
First visited non-NTP providers (n = 378)	
Pharmacy/drug shops	298 (79)
Traditional healer	38 (10)
Private clinics	38 (10)
Health posts	4 (1)
Reasons for not visiting a public facility first (n = 378)	
It was too expensive	83 (22)
It takes time	98 (26)
Long distance to the facility	87 (23)
Believed they would get better treatment elsewhere	68 (18)
Mistrust of public facility	38 (10)
Others	4 (1)

The overall median number of visits to HCPs before TB treatment was started was 3 (IQR 3, 5). The median number of HCP visits completed before initiation of treatment in the case of an initial non-NTP provider visit was 4 (IQR 3, 4) compared to 3 (IQR 2, 3) visits with an NTP provider (P = 0.001). This association remained significant after adjusting for age, gender, residence, and HIV (data not shown).

### Delay

The median patient delay was 8 (IQR 8, 12) weeks. In bivariate association, patient delay was significantly associated with older age, urban residence, illiteracy, longer walking distance to the nearest public facility, HIV seropositivity, and doing a chest X-ray (Table 4). The median reported health system delay was 3 (IQR 1, 4) weeks. Health system delay was significantly associated with older age, male gender, longer walking distance to the nearest public facility, first consulting a non-NTP provider, HIV seropositivity, and doing a chest X-ray (Table 4). The median total delay from onset of cough until start of anti-TB chemotherapy was 11 (IQR 9, 16) weeks. Total delay was significantly associated with older age, urban residence, illiteracy, longer walking distance to the nearest public facility, having sputum smear-negative TB, and doing a chest X-ray (Table 4). In all 312 (69%) of the subjects had prolong patient delay (i.e. > 4 weeks); 114 (25.3%) had prolonged health system delay (i.e. > median health system delay) and 216 (48%) had prolonged total delay (i.e. > median total delay).

**Table 4:** Tuberculosis treatment delays and its relationships with patients characteristics in rural Ebonyi State, 2011 (n = 450)

**Variables**	**Patient delay**		**HS delay**		**Total delay**	
	**Median (IQR)**	**P- value**	**Median (IQR)**	**P-value**	**Median (IQR)**	**P-value**
**Total**	8 (8, 12)		3 (1, 4)		11 (9, 16)	
**Prolonged delay N (%)**	312 (69%)		114 (25%)		216 (48%)	
**Age (years)**		0.02**		0.004**		<0.001**
≤40	8 (8, 12)		3 (2, 3)		11 (9, 15)	
41–65	12 (6, 20)		2 (1, 4)		14 (9, 25)	
>65	6 (6, 36)		4 (4, 4)		40 (40, 40)	
**Gender**		0.5		0.001**		0.6
Female	8 (8, 12)		2 (1, 3)		11 (9, 16)	
Male	8 (4, 12)		3 (2, 4)		12 (9, 16)	
**Residence**		0.04**		0.8		<0.001**
Rural	8 (6, 12)		3 (2, 3)		11 (9, 14)	
Urban	12 (10, 15)		3 (1, 4)		16 (11, 19)	
**Formal education**		0.006**		0.9		0.002**
Yes	8 (4, 12)		3 (1, 3)		11 (9, 16)	
No	9 (8, 12)		3 (2, 4)		13 (11, 16)	
**Walking distance to the** **nearest public facility**		0.015**		0.003**		<0.001**
< 60 minutes	8 (6, 8)		2 (1, 3)		11 (10, 14)	
60 – 119 minutes	8 (8, 12)		3 (2, 3)		10 (7, 14)	
>120 minutes	9 (8, 12)		4 (3, 4)		15 (11, 20)	
**First visited s non-NTP provider**		0.2		0.005		0.6
Yes	8 (8, 12)		3 (2, 4)		12 (10, 21)	
No	8 (5, 12)		2 (1, 3)		11 (9, 16)	
**Sputum smear status**		0.3		0.2		0.004**
Smear-positive	8 (8, 12)		3 (1, 4)		11 (9, 16)	
Smear-negative	8 (6, 12)		3 (2, 3)		15 (7, 25)	
**HIV status**		0.049**		0.02**		0.1
Positive	8 (6, 12)		3 (2, 4)		11 (9, 15)	
Negative	8 (8, 12)		2 (1, 3)		11 (9, 18)	
**Had a Chest X-ray**		<0.001**		0.01**		<0.001**
Yes	8 (8, 12)		3 (1, 4)		13 (9, 18)	
No	8 (4, 12)		2 (2, 3)		11 (7, 14)	

All delays are in weeks; HS = Health System; NTP = National Tuberculosis Control Programme; **P < 0.05.

### Determinants of delays

In multiple linear regression analysis, independent determinants of longer patient delay were older age (P <0.001), urban residence (P <0.001), longer walking distance to the nearest public facility (P <0.001), and doing a chest x-ray (P = 0.049); while HIV seropositivity (P = 0.011) and having a formal education (P = 0.036) were determinants of shorter delays (Table 5). Also, male gender (P = 0.004), and first visiting a non-NTP provider (P = 0.02) were found to be independent determinants of longer health system delay (Table 5). Furthermore, independent determinants of increased total delay were older age (P < 0.001), male gender (P =0.007), urban residence (P <0.001) and longer walking distance to the nearest public facility (0.03), (Table 5). An inspection of the graphs of the residuals suggests that the data for the delays met the assumptions for multiple linear regression analysis.

**Table 5:** Determinants of patient, health system and total delays among new pulmonary tuberculosis patients in rural Ebonyi State, 2011 (n = 450)

**Variables**	**β**	**SE**	**F-test**	**P-value**	**aR**^**2**^
**Patient delay**					
Constant	5.39	1.72	9.69	0.002	0.22
Older age	0.13	0.03	25.00	<0.001**	
Male gender	1.01	0.65	2.38	0.12	
Longer walking distance to the nearest public facility	0.014	0.003	18.50	<0.001**	
First visited a non-NTP provider	0.13	0.78	0.026	0.90	
HIV positive status	−1.77	0.69	6.50	0.011**	
Urban residence	3.65	0.84	19.00	<0.001**	
Had a chest x-ray done	1.35	0.69	3.90	0.049**	
Formally educated	−1.48	0.66	4.81	0.036**	
Smear-positive smear	−0.39	0.79	0.24	0.62	
**Health system delay**					
Constant	2.62	0.51	26.59	<0.001	0.08
Older age	0.008	0.007	1.09	0.30	
Male gender	0.52	0.18	8.18	0.004**	
Longer walking distance to a public facility	−0,001	0,001	2.75	0.10	
First visited a non- NTP provider	0.49	0.22	5.08	0.025**	
HIV positive status	0.36	0.19	3.65	0.06	
Urban residence	0.2	0.23	0.73	0.39	
Had a chest x-ray done	0.36	0.19	3.8	0.053	
Formally educated	0.29	0.18	2.66	0.10	
Sputum smear-positive	−0.39	0.21	3.09	0.08	
**Total delay**					
Constant	3.06	2.42	1.60	0.21	0.24
Older age	0.33	0.04	82.94	<0.001**	
Male gender	2.35	0.87	7.32	0.007**	
Longer walking distance to a public facility	0.009	0.004	4.70	0.031**	
First visited a non-NTP provider	0.50	1.04	0.23	0.63	
HIV positive status	−1.60	0.90	3.20	0.08	
Urban residence	4.26	1.11	14.64	<0.001**	
Had a chest x-ray done	−0.58	0.90	0.41	0.52	
Formally educated	−0.97	0.87	1.22	0.27	
Sputum smear-positive	−1.9	1.04	3.19	0.08	

(β) = coefficient, aR^2^ = adjusted correlation coefficient, **P < 0.05, SE = Standard error.

## Discussion

We examined the healthcare seeking behaviour of adult TB patients as well as treatment delays and its determinants in a low-resource setting with a high burden of TB/HIV. More than nine-tenth of the patients reported a walking distance of more than one hour to the nearest public healthcare facility to their homes. This reflects poor availability of public health facilities in rural settings in the state. In addition, it suggests that efforts should be made to expand and sustain such public health facilities. This will in turn form the bedrock in achieving the TB millennium development goal and universal health coverage [18].

Two-thirds of the patients in the study sought treatment more than four weeks after onset of symptoms, higher than in Malaysia (52%) [19], Iran (12%) [20], Ethiopia (41%) [21], Spain (43%) [22], Zambia (35%) [23] and the Philippines (50%) [24], but lower than in urban Nigeria (83%) [10]. This suggests that although the proportion of patients who sought treatment more than four weeks after onset of TB symptoms were higher in Nigeria compared to other countries, overall, with the current public enlightenment campaigns [12,13], there have been a reduction in the proportion of patients who delayed for more than a month before consulting the health system.

The median patient delay observed was consistent with findings from southwest Nigeria [10,11], but higher than the average patient delay of 31.7 days documented in low-and-middle income countries [25]. However, the median health system delay observed was lower than the average of 28.4 days found in low-and-middle income countries [25]. The total delay found in this study was longer than those reported in the Philippines and South Africa [23,26] but agrees with previous findings in high incidence settings [10,25]. Given the high coverage of DOTS in Nigeria, and the very low case detection rate [8], our findings suggest that TB patients face major barriers to care and many of them remain undetected.

Patients who first sought care at a NTP-provider made an average of 3 visits required by the diagnostic pathway compared to those who didn’t. The average number of visits made before commencing treatment after an initial visit to a non-NTP provider was higher because TB might not have been considered during the first visit to the informal providers, as a result; the patients might have been given inappropriate care which led to several other visits before reaching the appropriate health facility for TB care. However, median number of visits made irrespective of the source of care in this study were similar to what was found in South Africa [26], but less than what was found in Tajikistan and urban Zambia, where TB patients made on the average 4.8 and 6.7 visits respectively [27,28]. Additional strategies to shorten the interval between HCP visits in the diagnostic pathway may further reduce health system delay in our setting and improve TB control.

Factors associated with diagnostic and treatment delays have not always been replicated in all settings, populations, and may differ among countries [3-5]. In a systematic review of studies on TB diagnostic and treatment delay by Storla et al. [4], factors associated with delays in the health seeking pathway included HIV infection, older age, low educational level, longer walking distance to the nearest public facility, male gender, an initial visit to a non-NTP provider, rural residence and smear-negative sputum test; except for urban residence and HIV infection, these were consistent with our findings. The reason for higher delays among patients living in urban area may be because the study health facilities were located in the rural areas, and in order to probably avoid the stigma associated with TB care seeking in urban hospitals, they opted for care in a rural hospital. HIV positive patients had shorter patient delay probably because of the collaborative TB/HIV activities exiting in the health institutions which ensures early TB screening among HIV patients and vice versa [29].

To reduce delays in TB care in our setting, a high index of suspicion for TB needs to be maintained by all HCPs. To date, Nigeria has made several organised efforts to involve the private sector in TB care, through public-private mix initiatives [7]. These include involvement of non-governmental organisations, private (mission/for-profit) hospitals in TB care, which contributed to 39% of total TB case notification in 2010 [8]. Our study suggests that the next steps in this process would include involving pharmacy/drug shops and traditional healers in rural low-resource settings. There is a potential for earlier detection of TB in these patients if they would refer all coughing patients to a diagnostic clinic. In Malawi and Nigeria, drug shop owners and traditional healers have respectively indicated willingness to play a role in tuberculosis control [30,31].

Our study has several limitations. It was conducted in a rural, high TB/HIV under-resourced setting. Thus, our findings may not be generalisable to all settings. It would be valuable to obtain data on settings with different cultural and socio-economic characteristics in the future. A further limitation found in studies on TB treatment delays, is that they rely on patient recall. We reduced this by collecting data as soon as possible after the patient initiated TB treatment, and by helping them in their recall efforts. Another limitation is that we do not report on patient knowledge and stigma, and its effects on TB treatment delay. These factors have been shown to be associated with patient delay in other studies [3,4] and must be further investigated in order to design appropriate educational interventions for our setting.

## Conclusions

This study shows that poor patients living in a rural setting with high burden of TB and HIV frequently first access informal care providers for TB care before being diagnosed. This results in increased numbers of HCP visits, and long delays in the diagnosis and treatment of TB. Although the median total delay until start of treatment is generally high for most patients, specific and targeted interventions are needed for identified at-risk groups accounting for almost half the sample who exceeded the median delays. Further improved access to care, education of patients, engagement of informal care providers and strengthening of existing public-private models in TB control in such settings may impact positively on treatment delay and are therefore recommended.

## Abbreviations

TB: Tuberculosis; NTP: National tuberculosis control programme; DOTS: Directly observed treatment, short course; WHO: World health organisation; HCP: Healthcare provider.

## Competing interests

The authors declare that they have no competing interests.

## Authors’ contributions

KNU led the study from conception, planning to writing-up. IA contributed to designing the study and to writing up the manuscript. CON and ECO participated in design of the study, data collection, and analysis. All authors read and approved the final manuscript.

## Authors’ information

KNU is an Internal Medicine resident clinician and a facilitator for the National TB and Leprosy Control Programme in Ebonyi State interested in TB epidemiology. IA is a public health physician; the program officer of the National TB and Leprosy Control Programme in Ebonyi State and a deputy director in the Ebonyi State Ministry of Health – he co-ordinates TB control activities in the State. CON (an epidemiologist) and ECO are Family Medicine resident physicians at the Federal Teaching Hospital, Abakaliki, Ebonyi State, Nigeria.

## Pre-publication history

The pre-publication history for this paper can be accessed here:

http://www.biomedcentral.com/1472-6963/13/25/prepub
